# Mechanisms of Chimeric Cell Therapy in Duchenne Muscular Dystrophy

**DOI:** 10.3390/biomedicines12091996

**Published:** 2024-09-02

**Authors:** Maria Siemionow, Anna Ziemiecka, Katarzyna Bożyk, Krzysztof Siemionow

**Affiliations:** 1 Dystrogen Therapeutics Technology Polska sp. z o.o., 00-777 Warsaw, Poland; cieciuch@dystrogen.com (A.Z.); bozyk@dystrogen.com (K.B.); siemiok@dystrogen.com (K.S.); 2 Department of Orthopaedics, University of Illinois at Chicago, Chicago, IL 60612, USA; 3 Chair and Department of Traumatology, Orthopedics and Surgery of the Hand, Poznan University of Medical Sciences, 61-545 Poznan, Poland

**Keywords:** Dystrophin Expressing Chimeric (DEC) cells, mitochondrial transfer, chimeric mitochondria, DMD therapy, DEC mechanisms, Duchenne muscular dystrophy (DMD), DEC myogenic potential, dystrophin, myotube formation, chimeric cells

## Abstract

Despite scientific efforts, there is no cure for Duchenne muscular dystrophy (DMD), a lethal, progressive, X-linked genetic disorder caused by mutations in the dystrophin gene. DMD leads to cardiac and skeletal muscle weakness, resulting in premature death due to cardio-pulmonary complications. We have developed Dystrophin Expressing Chimeric (DEC) cell therapy, DT-DEC01, by fusing human myoblasts from healthy donors and from DMD patients. Preclinical studies on human DEC cells showed increased dystrophin expression and improved cardiac, pulmonary, and skeletal muscle function after intraosseous administration. Our clinical study confirmed the safety and efficacy of DT-DEC01 therapy up to 24 months post-administration. In this study, we conducted in vitro assays to test the composition and potency of DT-DEC01, assessing chimerism level and the presence of dystrophin, desmin, and myosin heavy chain. Myoblast fusion resulted in the transfer of healthy donor mitochondria and the creation of chimeric mitochondria within DT-DEC01. The Pappenheim assay confirmed myotube formation in the final product. This study highlights the unique properties of DT-DEC01 therapy and their relevance to DMD treatment mechanisms.

## 1. Introduction

Duchenne muscular dystrophy (DMD) is a genetic, progressive, neuromuscular disorder caused by mutations in the dystrophin gene, which is located on the X chromosome [1,2,3]. This condition results in the absence of dystrophin, a cytoplasmic protein that is vital for normal muscle function [4,5]. A lack of dystrophin leads to progressive muscle weakness, degeneration, and fibrosis, the last of which is ultimately responsible for cardiopulmonary dysfunction and the premature deaths of DMD patients [6,7,8]. There is no cure for DMD, and current treatment options focus on the alleviation of symptoms and management of complications [9,10,11]. Glucocorticoids, which slow the decline of muscle strength and function, are the only gold-standard therapy currently available for DMD patients [12,13,14,15]. Gene therapies are only effective in a limited number of DMD patients with specific gene mutations and are known to cause treatment-related adverse events [16,17,18,19]. Moreover, recently, phase 3 confirmatory clinical trials of new DMD therapies, such as exon-skipping and micro-dystrophin, are struggling to prove their efficacy over placebos [20,21,22].

Historically, allogeneic myoblasts from related or unrelated human donors have been used as a promising therapeutic approach for DMD [23,24,25,26]. However, despite encouraging preliminary results, low cell engraftment and myoblast rejection have presented major challenges. Consequently, immunosuppressive therapy was required to enhance engraftment and prevent myoblast rejection, which introduced another challenge due to the side effects associated with immunosuppression in DMD patients [27,28].

Therefore, it is clear that there is a need for newer and safer universal therapies for DMD patients. To address these needs and challenges, we have adapted the phenomenon of spontaneous myogenic cells fusion for therapeutic application aimed at regenerating DMD-affected muscles.

Myoblast cell fusion in vivo is regulated by molecular markers and results in the formation of myotubes and myofibers. This physiological process is crucial for maintaining muscle tissue homeostasis and repairing muscle tissue after injuries [29,30]. By applying our well-established, proprietary, ex vivo, and PEG-mediated myoblast cell fusion procedure, we have developed a new generation of Dystrophin Expressing Chimeric (DEC) cells based on the ex vivo fusion of human myoblasts derived from normal, healthy allogeneic donors and DMD patients [31].

Preclinical studies on *mdx* mouse models of DMD confirmed the long-term engraftment, biodistribution and safety of DEC therapy. Moreover, functional improvements in cardiac, diaphragm, and skeletal muscle strength and function correlated with a significant increase in dystrophin expression, which was observed after 90 and 180 days following systemic-intraosseous administration of DEC cells [32,33]. In addition, reduced *mdx* pathology correlated with improved muscle morphology and reduced fibrosis and inflammation, leading to an overall improvement in DMD-affected muscles after DEC therapy [34].

These studies introduced DEC as a novel cell-based therapeutic approach, which was further successfully tested on DMD patients in a proof-of-concept clinical study. The safety of DT-DEC01 therapy was confirmed by the absence of Adverse Events (AE) and Serious Adverse Events (SAE) up to 24 months after systemic-intraosseous DEC administration. Furthermore, DT-DEC01's efficacy was confirmed by improvements in cardiac and pulmonary function through echocardiography and spirometry, respectively [35]. Improved muscle strength correlated with an increased duration of the motor unit potentials (MUP) assessed by electromyography (EMG) [36]. It is important to note that these encouraging findings were observed in all DMD patients, regardless of gene mutation, age, or ambulatory status. Therefore, DEC can be considered as a universal therapy for all DMD patients, overcoming the limitations of other therapies that target only specific gene mutations.

In this study, the composition of patient-derived DT-DEC01 products was further assessed to support the mechanisms involved after systemic-intraosseous administration of DT-DEC01 therapy to DMD patients. Specifically, the in vitro assays confirmed the presence of dystrophin and myosin heavy chain in the created DT-DEC01 product. Additionally, myoblast fusion resulted in the transfer of healthy donor mitochondria and the creation of chimeric mitochondria within the DT-DEC01 product administered to DMD patients, as previously reported [37]. Furthermore, a Pappenheim assay confirmed that myotube formation was caused by the final DT-DEC01 product.

In summary, this study introduces DT-DEC01 as a novel, universal, Advanced Therapy Medicinal Product (ATMP) for all DMD patients, regardless of their gene mutation, age, or ambulatory status.

## 2. Materials and Methods

### 2.1. Creation of Human DT-DEC01 Product by PEG-Mediated Fusion of Myoblasts Derived from Normal, Healthy Donor and DMD Patient

Human muscle tissue samples were obtained from normal donors (*n* = 6) and DMD patients (*n* = 6) after obtaining informed consent for a first-in-human study (approval no. 46/2019, Bioethics Committee at the Regional Medical Council in Poznan, Poland).

Six pairs of myoblast cell lines from the donor and recipient were coded with numbers from 1 to 6, as MB^N1−6^ for the donors and MB^DMD1−6^ for the recipients, respectively.

Myoblast isolation and propagation as well as ex vivo PEG-mediated fusion of human myoblasts from normal, healthy donors (MB^N^) and DMD-patients (MB^DMD^), were performed as previously described [35]. Briefly, muscle tissue samples were isolated by enzymatic digestion with collagenase (Nordmark Pharma Gmbh, Uetersen, Germany) and cultured in a basal DMEM medium (HyClone) supplemented with ELAREM Ultimate—FDi human platelet lysate (PL Bioscience GmbH, Aachen, Germany), L-Alanyl-L-Glutamine (Biological Industries, Beit HaEmek, Israel), Antibiotic-Antimycotic (Gibco-ThermoFisher, Waltham, MA, USA), and human bFGF (Biotechne, Minneapolis, MN, USA).

The PEG-mediated ex vivo fusion was performed after harvesting the myoblast cells from both donors between passages 2 and 4. Next, the MB^N^ and MB^DMD^ cells were fluorescently labeled with PKH26 and PKH67 dyes (Sigma-Aldrich, St Louis, MO, USA), respectively. Then, both single-stained cell lines were fused with equal proportions in DMEM with 1.46 g/mL of polyethylene glycol (PEG 4000, Merck) and 16% DMSO (WAK-Chemie Medical GmbH, Steinbach, Germany). Finally, after fusion, cells were washed twice with a sorting buffer (Miltenyi Biotec, Teterow, Germany). The double-positive cells were sorted by a FACS MACSQuant Tyto sorter (Miltenyi Biotec) and propagated until obtaining a sufficient number of cells for the preparation of personalized DT-DEC01 products for systemic-intraosseous administration to the DMD patients, as previously described [35].

### 2.2. Assessment of Donor-Specific Chimerism by STR-PCR Analysis of Donor Specific STR Loci

The final DT-DEC01 product was analyzed for the presence of donor-specific chimerism by a polymerase chain reaction short-tandem repeat (STR-PCR) analysis. Batches of patient-derived DT-DEC01 products were analyzed before administration to the DMD patients, as previously described [35]. Briefly, after isolating DNA from MB^N^, MB^DMD^, and DEC cells, DNA amplification and electrophoresis were performed. The data was processed using ChimerMaker software v. 3.1.8 (SoftGenetics LLC, State College, PA, USA), focusing on specific loci. The STR profile was determined for the donor, the DMD patient, and the created DT-DEC01 product. Donor chimerism was assessed by analyzing informative loci to determine the percentage of the donor STR loci in DT-DEC01 products.

### 2.3. Characterization of the Human DT-DEC01 Product In Vitro by Immunofluorescence Detection of Desmin, Dystrophin and Myosin Heavy Chain

To characterize the myogenic potential of each personalized DT-DEC01 product prepared for systemic-intraosseous administration to DMD patients, the parent cell lines of myoblasts from a normal, healthy donor (MB^N^) and a DMD patient (MB^DMD^) were analyzed before cell fusion. The DT-DEC01 products, created by PEG-mediated fusion of the parent cell myoblasts, were analyzed after cell fusion. To determine the myogenic potential of the parent and DEC cells, immunofluorescence analyses were conducted to detect the expression of desmin (DES), dystrophin (DYS), and myosin heavy chain (MyHC).

The presence of the myogenic marker of desmin (DES) was confirmed in the undifferentiated cells approximately 4 days after cell seeding. To confirm the presence of myogenic markers of dystrophin (DYS) and myosin heavy chain (MyHC), all cell lines were subjected to the differentiation process as described below. After up to 21 days of differentiation, cells were fixed with cold 4% PFA (15 min), washed, permeabilized in 0.1% Triton (Sigma-Aldrich, 15 min), and blocked overnight (4 °C) in a blocking buffer containing 5% albumin (CSL Behring) and 0.5% Triton. After washing, cells were incubated overnight (4°C) in a blocking buffer with the primary antibodies of mouse-monoclonal anti-human desmin antibody (1:200; cat. D1033, Sigma-Aldrich), mouse-monoclonal anti-human dystrophin antibody (1:100; cat. D8168, Sigma-Aldrich), and mouse-monoclonal anti-human myosin (skeletal, slow) antibody (1:100; cat. M8421, Sigma-Aldrich), then washed in PBS (VWR) and incubated in the dark for 60 min (RT) with Alexa Fluor 488 conjugated donkey anti-mouse IgG (H + L) secondary antibody (1:500, cat. A-21202, Invitrogen, Waltham, MA, USA) in PBS (VWR). Nuclei were counterstained with DAPI Fluoroshield (Sigma Aldrich). A Leica DMi8 microscope was used for fluorescence signal detection.

### 2.4. Assessment of the Intercellular Mitochondrial Transfer and Mitochondrial Chimeric State in the Created DT-DEC01 Product

To further support the mechanism of action of DEC cells, mitochondrial fusion and mitochondrial transfer were assessed in DEC cells after ex vivo PEG-mediated fusion [37]. Myoblasts from normal, healthy donors (MB^N^) and DMD patients (MB^DMD^), isolated during the ATMP-HE Therapeutic Experiment, were fluorescently labeled using MitoTracker DeepRed and MitoTracker Green mitochondrial dyes (Sigma), respectively.

The cell samples from normal and DMD donors were analyzed to confirm mitochondrial fusion and transfer flow cytometry (FACSLyric, BD Biosciences, San Jose, CA, USA). The excitation and emission wavelengths for MTDeepRed were set at 633 nm and >650 nm, and for MTGreen, they were set at 488 nm and 505–570 nm, respectively. Briefly, cells were attached to the polylysine-coated slides, fixed, counterstained with DAPI, and sealed with a mounting medium. The co-localization of MTDeepRed and MTGreen fluorescent dyes within the cells was assessed in FarRed and Green channels, respectively.

### 2.5. Characterization of the Human DT-DEC01 Product In Vitro by Detection of Myotube Formation

To assess the potential of the created personalized DT-DEC01 products towards myoblast fusion and myotube formation after administration, an in vitro analysis was performed on the final DT-DEC01 products. For the assessment of myotube formation, the parent cells of each normal, healthy donor and DMD patient, as well as the DEC cell lines created from them, were cultured until the myoblasts reached around 90% confluence. Next, the culture medium was changed to a differentiation medium [either 2% Horse Serum (Biowest, Nuaillé, France) in basal DMEM medium, or Skeletal Muscle Differentiation Medium (Promocell, Heidelberg, Germany)], and the cells were cultured for up to 21 days.

Next, the cells were fixed (cold methanol, 3 min) to be assessed for myotube formation via Pappenheim staining. May-Grunwald (Sigma-Aldrich) dye was added during a 3 min incubation (RT), followed by staining with 5% Giemsa (Sigma-Aldrich) solution for 20 min (RT). Samples were washed once in PBS (VWR) and imaged under a light microscope (Nikon Eclipse Ts2, Nikon Instruments Inc., Melville, NY, USA).

## 3. Results

**Figure 1:**
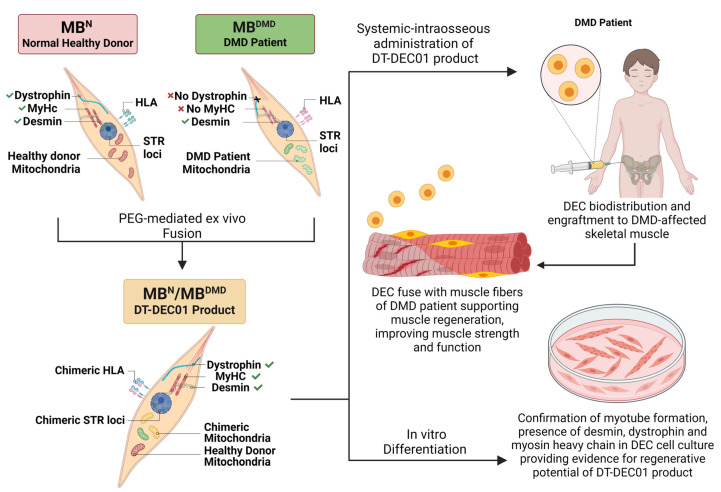
Confirmation of the creation of patient-derived DT-DEC01 product by PEG-mediated fusion of myoblasts derived from a normal, healthy donor and a DMD patient, and the mechanisms of action of DT-DEC01 after intraosseous administration to a DMD patient. The patient-derived DT-DEC01 product was created by PEG-mediated fusion of myoblasts from a normal, healthy donor (MB^N^) and from a DMD patient (MB^DMD^). Before fusion, the presence of dystrophin (DYS), myosin heavy chain (MyHC) and desmin (DES) was confirmed in the myoblasts of normal, healthy donor (MB^N^) origin; however, there was a lack of expression of DYS and MyHC in the myoblasts of DMD patients (MB^DMD^). In addition, the cells were subjected to an analysis of their STR loci. After PEG-mediated fusion, the presence of DES, DYS, and MyHC expression was confirmed in the created patient-derived MB^N^/MB^DMD^ DT-DEC01 product, indicating the delivery of the proteins essential for the enhancement of muscle strength and regeneration after the systemic-intraosseous administration of DT-DEC01 product into the DMD patient. Moreover, the PEG-mediated myoblast fusion resulted in the transfer of healthy donor mitochondria and the formation of chimeric mitochondria within the created patient-derived DT-DEC01 product, suggesting the enhanced therapeutic potential of DT-DEC01 therapy in restoration of a mitochondrial function in the DMD-affected muscles. The presence of donor-recipient chimerism in the created MB^N^/MB^DMD^ final DT-DEC01 product was confirmed by detecting the STR loci of both the donor and the recipient. In vitro differentiation confirmed myotube formation by the created MB^N^/MB^DMD^ cells, providing evidence for myogenic differentiation and regenerative potential of the patient-derived DT-DEC01 product after systemic administration to DMD patients. In summary, systemic-intraosseous administration of DT-DEC01 resulted in the biodistribution and engraftment of MB^N^/MB^DMD^ cells to the DMD-affected skeletal muscles, where MB^N^/MB^DMD^ cells fuse with the muscle fibers of DMD patient and are expected to deliver dystrophin—the protein responsible for enhancement of regeneration and restoration of muscle strength and function.

The creation of patient-derived DT-DEC01 products through the PEG-mediated fusion of PKH single-stained myoblasts from a healthy donor (MB^N^) and DMD patient (MB^DMD^), as well as the mechanisms involved in DMD following systemic-intraosseous administration of the DT-DEC01 product, are summarized in Figure 1.

### 3.1. Confirmation of Creation of Patient-Derived Chimeric (MB^N^/MB^DMD^) DT-DEC01 Product by PEG-Mediated Fusion of Myoblasts from Normal, Healthy Donor and DMD Patient

**Figure 2:**
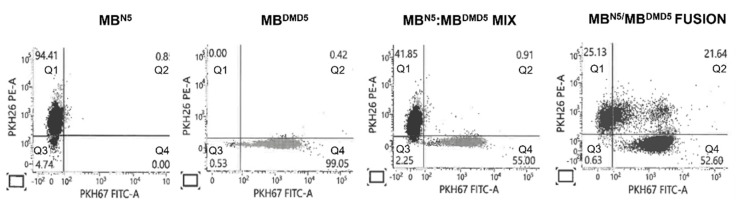
Confirmation of creation of Dystrophin Expressing Chimeric (DEC) cells derived from normal, healthy donors (MB^N5^) and DMD patients (MB^DMD5^). The representative flow cytometry dot plots confirm the fusion of the MB^N5^ and MB^DMD5^ myoblasts stained with the PKH26 and PKH67 dyes, respectively. Starting from the left, the plots show the fluorescence analysis of PKH26 (Q1) and PKH67 (Q4) staining, next the MIX of the single-stained MB^N5^-PKH26 and MB^DMD5^-PKH67 (Q1 and Q4, respectively) cells, and at the far right, the overlapping of PKH26/PKH67 (Q2) dyes confirming the creation of the patient-derived, donor-recipient chimeric myoblasts (MB^N5^/MB^DMD5^) representing DT-DEC01 product.

A flow cytometry analysis of normal donor (MB^N5^) myoblasts stained with PKH26 dye and PKH67-stained DMD patient (MB^DMD5^) myoblasts confirmed staining efficacies of 94.41% and 99.05%, respectively. The efficacy of PEG-mediated fusion was revealed to be 21.64% and was based on the overlapping of both PKH dyes (PKH26/PKH67), confirming the presence of the donor-recipient chimeric state (Figure 2).

### 3.2. Confirmation of Donor-Specific Chimerism in Patient-Derived DT-DEC01 Product through Detection of the STR Loci Specific for the Normal Myoblast Donor

The donor-specific chimerism in the final, patient-derived DT-DEC01 products was further confirmed by a polymerase chain reaction short-tandem repeat (STR-PCR) analysis. A quantitative analysis of the STR loci profiling in the final DT-DEC01 products revealed, in each tested batch, the presence of STR loci corresponding with the parent cell lines derived from both the normal donors (MB^N^) and DMD patients (MB^DMD^) (Table 1). The assessed donor-specific chimerism ranged between 6–35% and was confirmed by the presence of STR loci specific for normal myoblast donors (MB^N^) in the final DT-DEC01 products.

### 3.3. Confirmation of Myogenic Potential of the Patient-Derived DT-DEC01 Product through Expression of Desmin, Dystrophin and Myosin Heavy Chain

**Figure 3:**
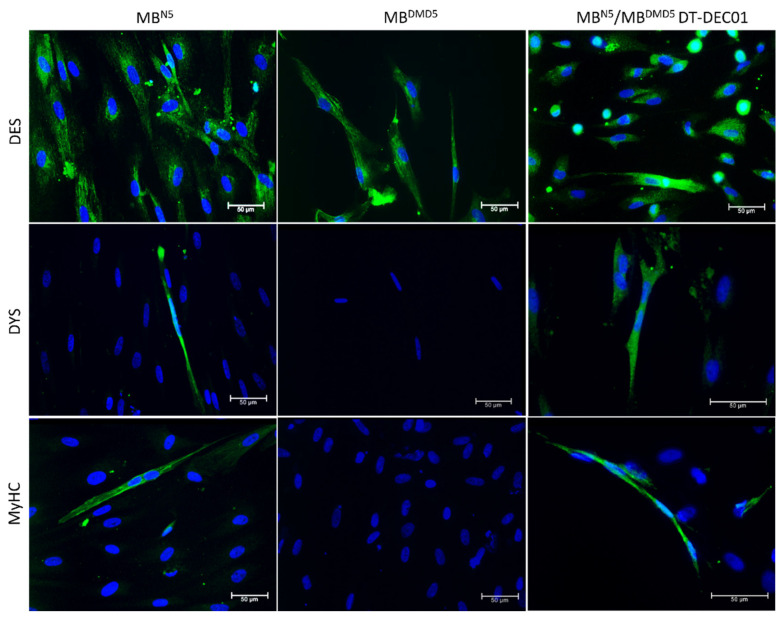
Detection of the expression of desmin, dystrophin and myosin heavy chain in the cell line of Normal Donor MB^N5^ and the cell line of DMD Patient MB^DMD5^ before cell fusion, and in the DT-DEC01 product after parent cell fusion. The expression of desmin (DES), dystrophin (DYS), and myosin heavy chain (MyHC) (green) is shown on the representative immunofluorescence images of the donor MB^N5^ cell line (**first column**). The presence of desmin was also confirmed in the MB^DMD5^ cell line of the DMD patient, however there was a lack of expression of DYS and MyHC observed in the cell line of MB^DMD5^ patient (**second column**). The expression of all three markers (DES, DYS, MyHC) was confirmed in the MB^N5^/MB^DMD5^ DT-DEC01 product after parent cell fusion (**third column**). Nuclei were counterstained with DAPI (blue) in each cell line. DES was detected in undifferentiated cells. DYS and MyHC were detected in myotubes following the 8-day differentiation process in Promocell medium. The magnification of images on the panel is 40×, and a scale bar is 50 μm. For the images of DYS and MyHC presence in MB^N5^/MB^DMD5^ DT-DEC01 magnification is 64× and scale bar 50 μm.

The immunofluorescence analyses of cell lines isolated from muscle biopsies of healthy donors and DMD patients confirmed the presence of 100% desmin (DES) expression before cell fusion. Furthermore, the DEC cells created by the fusion of the parent cell lines confirmed 100% DES expression after fusion (Figure 3).

Furthermore, immunofluorescence analyses performed before cell fusion confirmed the expression of dystrophin (DYS) and myosin heavy chain (MyHC) in the cell culture of the normal, healthy donors, whereas there was a lack of DYS and MyHC expression in the cell culture of DMD patients. However, after parent cell fusion, dystrophin (DYS) and myosin heavy chain (MyHC) was expressed in the fused DEC cells, confirming the myogenic potential and delivery of the full-length dystrophin of the healthy donor origin within the patient-derived final DT-DEC01 product (Figure 3).

### 3.4. Confirmation of Transfer of Healthy Donor Mitochondria and Presence of Chimeric Mitochondria in the Patients-Derived DT-DEC01 Product

**Figure 4:**
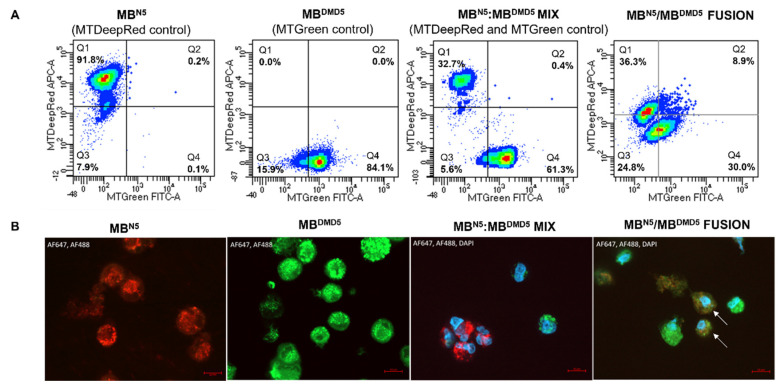
Confirmation of mitochondrial transfer and creation of chimeric mitochondria via ex vivo PEG-mediated fusion of myoblasts derived from normal and DMD-affected human donors. (**A**) Representative flow cytometry dot plots confirming mitochondrial fusion of the MB^N5^/MB^DMD5^ cells stained with the MTDeepRed or MTGreen dyes, assessed by FACS. Starting from the right, the plots show the fluorescence analysis of MTDeepRed (Q1) and MTGreen (Q4) mitochondria, the MIX of the single-stained MB^N5^ MTDeepRed, the MB^DMD5^ MTGreen mitochondria (Q1 and Q4), and the overlapping fluorescence of MTDeepRed/MTGreen (Q2), confirming the chimeric state of the mitochondria. Panel (**A**) modified from Siemionow et al., 2024 [37] based on the copyrights owned by the authors. (**B**) Representative confocal microscopy images of the myoblasts before and after fusion and in the merged channels. The images show the control of the single-stained MB^N5^ mitochondria with MTDeepRed (red); the MB^DMD5^ mitochondria stained with MTGreen (green); the MIX of the the single-stained MTDeepRed and MTGreen myoblasts; and the DEC cells after fusion. Nuclei were counterstained with DAPI (blue). The white arrows indicate chimeric cells with the overlapping signals of MTDeepRed/MTGreen, indicating the chimeric mitochondria (magnification 63×, scale bar 10 μm).

Following the staining procedure, PEG-mediated fusion was performed using myoblasts from a normal, healthy donor (MB^N5^) and a DMD patient (MB^DMD5^). The fusion efficacy of the myoblasts reached 8.83%. The created chimeric MB^N5^/MB^DMD5^ DEC cells exhibited mitochondria of normal donor origin, stained with the MTDeepRed, and the mitochondria of DMD patient origin, stained with the MTGreen dye, indicating mitochondrial transfer and fusion, as previously reported [37] (Figure 4A).

Furthermore, colocalization of the MTDeepRed and MTGreen dyes in the created MB^N5^/MB^DMD5^ DT-DEC01 product confirmed intracellular transfer and mitochondrial chimerism within the fused myoblasts. A confocal microscopy analysis revealed the presence and the overlap of the fluorescence staining of MTDeepRed/MTGreen dyes, confirming the mitochondrial transfer and the chimeric mitochondria in the MB^N5^/MB^DMD5^ DEC DT-DEC01 product (Figure 4B).

### 3.5. Confirmation of Myogenic Potential of the Patient-Derived DT-DEC01 Product by Detection of Myotube Formation

**Figure 5:**
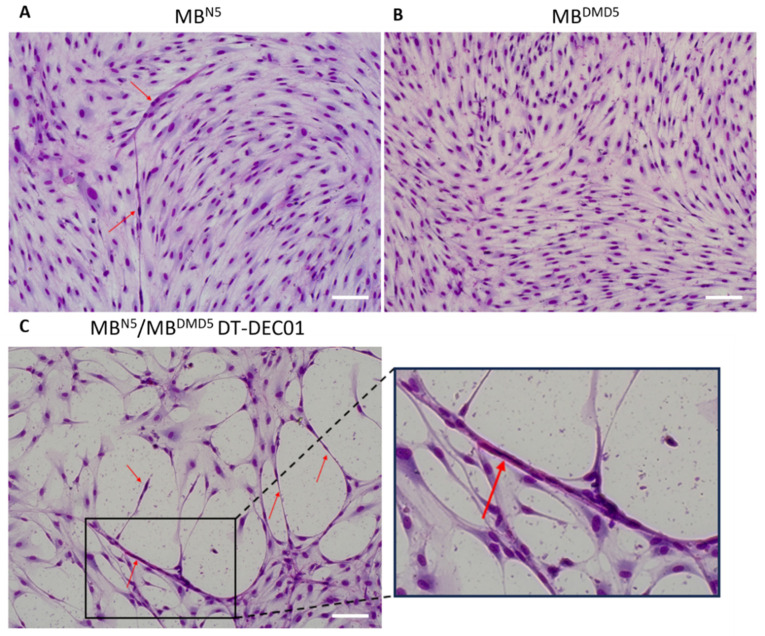
Detection of myotube formation in the cell lines of (**A**) myoblasts derived from normal, healthy donor (MB^N5^) and (**B**) myoblasts derived from the DMD patient (MB^DMD5^) before cell fusion and (**C**) in the patient-derived MB^N5^/MB^DMD5^ DT-DEC01 product after parent cell fusion. Assessment of the myotube formation is shown on the representative Pappenheim staining (May-Grunwald/Giemsa method) images of the (**A**) cell line of the normal, healthy donor MB^N5^ (**upper left**), (**B**) cell line of the DMD patient MB^DMD5^ (**upper right**) and (**C**) the MB^N5^/MB^DMD5^ DT-DEC01 drug product after parent cells fusion (**lower left** and **right**). The presence of myotubes before fusion was detected in the normal donor cell line (MB^N5^), however, myotubes were absent in DMD patient cell line (MB^DMD5^). The formation of multiple myotubes was confirmed in the created MB^N5^/MB^DMD5^ DT-DEC01 product. Pappenheim stainings were assessed following the (**A**,**B**) 8-day and (**C**) 21-day differentiation process in 2% HS medium. Magnification of images on the panel: 200×, scale bar: 100 μm; the lower right image of MB^N5^/MB^DMD5^ DT-DEC01 magnification: 440×. Arrows indicate examples of multinuclear myotubes.

An immunofluorescent assessment of differentiation and a Pappenheim staining analysis revealed the lack of myotube formation by the DMD patient cell lines after 21 days of culture in the differentiation medium (Figure 5). In contrast, both the healthy donor cells and the fused DEC cells revealed the presence of dystrophin (DYS) and myosin heavy chain (MyHC) and confirmed myotube formation, providing evidence for myogenic differentiation and the regenerative potential of the created patient-derived DT-DEC01 Dystrophin Expressing Chimeric (DEC) cells.

## 4. Discussion

Currently, there is a lack of effective therapies for Duchenne muscular dystrophy patients, and new therapeutic approaches encounter significant challenges due to safety concerns and their lack of proven efficacy [38,39]. For gene therapies, these challenges include the risks associated with off-site mutations, tumorigenicity, and development of sensitization [40]. Moreover, a major problem is their lack of expected efficacy; as reported recently in phase 3 confirmatory clinical trials of emerging DMD therapies, including exon-skipping and micro-dystrophin, they failed to demonstrate favorable efficacy versus placebo treatment [21,22]. Furthermore, gene therapies targeting a specific gene mutation are effective only for a limited population of DMD patients [41,42]. Similar concerns are posed for ataluren, designated for an estimated 10% of the population of DMD patients with nonsense mutations [43,44,45]. Based on scientific and clinical reports, it is evident that DMD is a complex disease caused by various gene mutations. This represents the critical challenge when enrolling DMD patients for treatments with different gene therapies [46]. Despite scientific effort, DMD patients currently still lack access to a universal and effective therapy. To address these challenges, we introduce DT-DEC01, or Dystrophin Expressing Chimeric (DEC) cell therapy, as an alternative universal therapeutic approach that overcomes the limitations of current DMD treatments [47,48,49]. As a result, DT-DEC01 therapy has many potential applications for the entire population of DMD patients.

During pre-clinical studies, tested in *mdx* mouse models of DMD as well as during clinical development of DEC cell–based therapy, we have demonstrated that DEC creation does not require cellular reprogramming, genome editing, or viral vector-induced engineering, and as such has the potential to be safer [31,32,33,34]. Thus, the proposed DT-DEC01 therapeutic approach, based on donor (MB^N^)–recipient (MB^DMD^) chimeric cells, is unique and has the potential to become a universal and global therapy applicable to all DMD patients, regardless of whichever gene mutation causes the disease.

This study validates the therapeutic potential of personalized, patient-derived DT-DEC01 products composed of Dystrophin Expressing Chimeric (DEC) cells for the treatment of Duchenne muscular dystrophy (DMD). Our data provide substantial evidence for the efficacy and myogenic potential of DT-DEC01 through several key findings summarized below.

The high efficacy of staining with PKH dyes and successful PEG-mediated fusion highlight the efficacy and reliability of the cell fusion technique [50,51]. This was further confirmed by the creation of donor–recipient chimeric cells, representing a critical step for ensuring the therapeutic integration of healthy donor cells with those derived from the DMD patient. The ex vivo fusion process is essential for the intended therapeutic effects, as it combines the healthy donor’s cellular machinery with the patient’s cells, facilitating regeneration and restoration of function in dystrophic muscle tissues.

Our study confirmed donor-specific chimerism in the patient-derived DT-DEC01 product by identifying the short tandem repeat (STR) loci specific to the normal myoblast donors. The detection of these genetic markers indicated the successful incorporation and maintenance of donor cell characteristics within the patient-derived product. Donor-specific chimerism is essential for ensuring the therapeutic efficacy and safety of the engrafted cells, as it signifies that the donor-recipient chimeric DEC cells are not only recognized as the patient's own cells, but also retain beneficial properties of the cells of a normal, healthy donor. Furthermore, the delivery of donor-specific chimerism within the DT-DEC01 product enhances engraftment and prevents cell rejection without the need for immunosuppression, as confirmed in our clinical study [35].

The presence of desmin, dystrophin, and myosin heavy chain in the fused, patient-derived DT-DEC01 product underscores the myogenic potential of DEC cells [52,53,54]. Desmin, a muscle-specific intermediate filament protein, is a crucial component in maintaining the structural unity and function of muscle cells [55]. The detection of dystrophin, which was absent in the myoblasts of DMD patients before DT-DEC01 administration, is particularly significant, as it proves that DEC cells provide functional dystrophin after administration to the DMD patients with the intent of restoring dystrophin in the DMD-affected muscles, thus addressing the primary cause of DMD [4]. Additionally, the presence of myosin heavy chain, a marker of muscle cell differentiation, further corroborate the capability of DT-DEC01 cells to undergo myogenic differentiation [56].

Furthermore, this study demonstrated successful mitochondrial transfer and mitochondrial chimerism within the fused DEC cells. The presence of mitochondria from the cells of both healthy donors and DMD patients within the chimeric DEC cells suggests a potential mechanism for restoring mitochondrial function in DMD-affected muscles. The mitochondrial transfer could alleviate some of the metabolic dysfunctions associated with DMD, thereby contributing to muscle repair and regeneration [57,58,59,60]. The ability to transfer healthy mitochondria from donor cells to patient cells is particularly beneficial because mitochondrial dysfunction is recognized as a key factor in the progression of DMD [61,62,63]. By introducing functional mitochondria, DT-DEC01 will enhance cellular energy production, reduce oxidative stress, and improve overall muscle cell health and function. This aspect of the therapy addresses both the primary genetic defect and the secondary metabolic deficiencies in DMD.

Furthermore, the formation of myotubes by the differentiated DEC cells after fusion, as opposed to the patient-derived cells alone, indicates their enhanced regenerative capacity. Myotube formation is a key step in muscle regeneration, reflecting the cells’ ability to differentiate into mature muscle fibers capable of contracting and generating force [64]. The observed myotube formation in DEC cells provides strong evidence for their myogenic differentiation capabilities, further supporting the therapeutic potential of the DT-DEC01 product.

The intraosseous administration of DEC cells is advantageous, as it facilitates efficient delivery and widespread distribution of the therapeutic cells throughout the entire body with better cell engraftment and safety profiles, but without causing a risk of embolism when compared to intravenous transplantation [65,66,67,68,69,70]. In contrast to local administration, which limits the efficacy of the injected cells only to the injected tissues [24,25,26,71,72], intraosseous injection into the bone marrow allows chimeric DEC cells to enter systemic circulation [73,74,75]. This enables them to converge on and repair damaged muscle tissues in various organs affected by DMD [31,32,33,34]. This method maximizes the therapeutic reach of the cells and enhances the overall effectiveness of the treatment.

We acknowledge some limitations of the study. The patient-derived DT-DEC01 products were manufactured for a small group of DMD patients in a proof-of-concept clinical study. It should be emphasized, however, that since DMD is a rare disease, it is often challenging to collect extensive data due to the limited size of the patient population and, consequently, the number of products that can be created. Therefore, in this study, we focused on providing new findings and unique characteristics of DT-DEC01 products tested in this first-in-human clinical study. We admit that for a more comprehensive correlation between predictive clinical outcomes and the potency of DT-DEC01 products, future clinical studies in a larger population of DMD patients are warranted.

Importantly, despite these limitations, the presented findings confirm the unique properties of DT-DEC01 therapy as a universal therapeutic approach for all DMD patients, and they underscore the importance of the presented treatment mechanisms in Duchenne muscular dystrophy.

## 5. Conclusions

This study assessed the composition and potency of the personalized, patient-derived DT-DEC01 product, correlating the in vitro tests with the mechanisms associated with systemic-intraosseous DEC administration to DMD patients. Key findings included the confirmation of the tolerogenic properties of DEC through a high level of chimerism, evidenced by the presence of donor-specific STR loci in the DEC product administered to the DMD patients. Additionally, the detection of dystrophin, desmin, and myosin heavy chain, along with the successful transfer of healthy donor mitochondria, underscores the regenerative potential of DT-DEC01 therapy. Moreover, effective myotube formation was demonstrated, further confirming the DT-DEC01 product’s capability for muscle regeneration.

Overall, this study presents, for the first time, the unique properties of personalized chimeric DT-DEC01 therapy created through the fusion of myoblasts from healthy donors and DMD patients. It also highlights its relevance to DMD treatment mechanisms, paving the way for future clinical applications aimed at restoring dystrophin and improving functional outcomes for all DMD-affected patients regardless of gene mutation, age, or ambulatory status.

## Figures and Tables

**Table 1:** Confirmation of donor-specific chimerism in patient-derived DT-DEC01 products by the presence of STR loci specific to myoblasts from normal, healthy donors (MB^N^).

ID of the MB^N^/MB^DMD^ DT-DEC01 Product	Donor (MB^N^)-Specific STR Loci
MB^N1^/MB^DMD1^	27%
MB^N2^/MB^DMD2^	21%
MB^N3^/MB^DMD3^	6%
MB^N4^/MB^DMD4^	35%
MB^N5^/MB^DMD5^	15%
MB^N6^/MB^DMD6^	6%

## Data Availability

The original contributions presented in the study are included in the article, further inquiries can be directed to the corresponding author.
